# When are pathogen genome sequences informative of transmission events?

**DOI:** 10.1371/journal.ppat.1006885

**Published:** 2018-02-08

**Authors:** Finlay Campbell, Camilla Strang, Neil Ferguson, Anne Cori, Thibaut Jombart

**Affiliations:** 1 MRC Centre for Outbreak Analysis and Modelling, Department of Infectious Disease Epidemiology, School of Public Health, Imperial College London, London, United Kingdom; 2 Centre for Preventive Medicine, Department of Epidemiology and Disease Surveillance, Animal Health Trust, Suffolk, United Kingdom; Cornell University, UNITED STATES

## Abstract

Recent years have seen the development of numerous methodologies for reconstructing transmission trees in infectious disease outbreaks from densely sampled whole genome sequence data. However, a fundamental and as of yet poorly addressed limitation of such approaches is the requirement for genetic diversity to arise on epidemiological timescales. Specifically, the position of infected individuals in a transmission tree can only be resolved by genetic data if mutations have accumulated between the sampled pathogen genomes. To quantify and compare the useful genetic diversity expected from genetic data in different pathogen outbreaks, we introduce here the concept of ‘transmission divergence’, defined as the number of mutations separating whole genome sequences sampled from transmission pairs. Using parameter values obtained by literature review, we simulate outbreak scenarios alongside sequence evolution using two models described in the literature to describe transmission divergence of ten major outbreak-causing pathogens. We find that while mean values vary significantly between the pathogens considered, their transmission divergence is generally very low, with many outbreaks characterised by large numbers of genetically identical transmission pairs. We describe the impact of transmission divergence on our ability to reconstruct outbreaks using two outbreak reconstruction tools, the R packages *outbreaker* and *phybreak*, and demonstrate that, in agreement with previous observations, genetic sequence data of rapidly evolving pathogens such as RNA viruses can provide valuable information on individual transmission events. Conversely, sequence data of pathogens with lower mean transmission divergence, including *Streptococcus pneumoniae*, *Shigella sonnei* and *Clostridium difficile*, provide little to no information about individual transmission events. Our results highlight the informational limitations of genetic sequence data in certain outbreak scenarios, and demonstrate the need to expand the toolkit of outbreak reconstruction tools to integrate other types of epidemiological data.

## Author summary

The increasing availability of genetic sequence data has sparked an interest in using pathogen whole genome sequences to reconstruct the history of individual transmission events in an infectious disease outbreak. However, such methodologies rely on pathogen genomes mutating rapidly enough to discriminate between infected individuals, an assumption that remains to be investigated. To determine pathogen outbreaks for which genetic data is expected to be informative of transmission events, we introduce here the concept of ‘transmission divergence’, defined as the number of mutations separating pathogen genome sequences sampled from transmission pairs. We characterise transmission divergence of ten major outbreak causing pathogens using simulations and find significant variation between diseases, with viral outbreaks generally exhibiting higher transmission divergence than bacterial ones. We reconstruct these outbreaks using the R-packages *outbreaker* and *phybreak* and find that genetic sequence data, though useful for rapidly evolving pathogens, provides little to no information about outbreaks with low transmission divergence, such as *Streptococcus pneumoniae* and *Shigella sonnei*. Our results demonstrate the need to incorporate other sources of outbreak data, such as contact tracing data and spatial location data, into outbreak reconstruction tools.

## Introduction

Understanding transmission dynamics in the early stages of an infectious disease outbreak is essential for informing effective control policy. Valuable insights can be gained by the reconstruction of the transmission tree, which describes the history of infectious events at the resolution of individual cases [1–4]. Recent years have seen significant progress in the development of statistical and computational tools for inferring such trees [5–15], with a major emphasis placed on the analysis of whole genome sequence (WGS) data, now routinely collected in many outbreak scenarios [16].

Two approaches to the inference of transmission trees from WGS have emerged. One begins with an underlying transmission model, attaching to this a model of sequence evolution that relates observed genetic relationships between pathogens to unobserved epidemiological relationships (*i*.*e*. transmission pairs) between infected individuals. A simple implementation involves ruling out direct transmission events between individuals separated by more than a fixed threshold of substitutions [17–19]. More sophisticated methods have specified models of sequence evolution as components in a joint likelihood, formalising expected genetic relationships in a probabilistic manner [5–9,20]. The other approach considers outbreak reconstruction from a phylogenetic perspective, inferring unobserved historical relationships between pathogen samples to capture more complex evolutionary dynamics. WGS data is used to reconstruct phylogenetic trees which are either treated as data upon which transmission histories are overlaid [10–12], or jointly inferred alongside the transmission tree itself [13–15]. Given the unprecedented level of detail of WGS data and the epidemiological insights it has provided in real-life scenarios [21–23], genetic analysis is clearly an indispensable tool for outbreak reconstruction.

However, a fundamental and so far largely unaddressed limitation of WGS data in informing outbreak reconstruction is the requirement for genetic diversity to accumulate on epidemiological timescales. The scope of outbreak scenarios for which such requirements are met has, to our knowledge, never been described. Specifically, at least one mutation must accumulate in the time between sampling of two individuals in a given transmission pair (i.e., an infector and a secondary case) in order for their position within the transmission tree to be distinguishable by genetic means. This represents a limit in the resolution of the data itself, independent of the methodology considered. Though groups of genetically identical pathogens may be identified as a cluster of infections, finer reconstruction of the transmission events would be impossible based on genetic data alone. Such limitations may be even more problematic in methods relying on accurately estimated phylogenetic trees for inferring transmission events [11,14].

The impact of limited genetic diversity on the reconstruction of disease outbreaks remains to be investigated. While this impact undoubtedly varies across different methods, the intrinsic informativeness of genetic data with respect to the underlying transmission tree can be evaluated. The genetic diversity accumulating along transmission chains depends on various genomic and epidemiological factors. To quantify this diversity, we introduce the concept of ‘*transmission divergence*’, which we define as the number of mutations accumulating between pathogen WGS sampled from transmission pairs.

Transmission divergence can be estimated empirically from a transmission tree by determining the number of mutations separating pathogen samples of known transmission pairs. However, accurately reconstructed transmission trees with corresponding genetic sequence data are generally not available for most pathogens. We present here a simulation based approach for estimating the transmission divergence of different pathogens using parameters available in the literature, namely the length of the pathogen genome (*L*), its overall mutation rate (*M*), its generation time distribution (*W*) (*i*.*e*. the distribution of delays between primary and secondary infections [24]) and its basic reproduction number R_0_ (i.e. the average number of secondary infections caused by an index case in a fully susceptible population [25]). Specifically, we model transmission trees alongside sequence evolution and extract the number of mutations separating individual transmission pairs. Intuitively, greater transmission divergence should enable better reconstruction of these transmission trees, although the nature of this relationship remains to be described.

To explore this issue, we compare the transmission divergence of ten major outbreak-causing pathogens, namely *Zaire ebolavirus* (EBOV), SARS coronavirus (SARS-CoV), MERS coronavirus (MERS-CoV), pandemic influenza A (H1N1), Methicillin-Resistant *Staphylococcus aureus* (MRSA), *Klebsiella pneumoniae (K*. *pneumoniae)*, *Streptococcus pneumoniae (S*. *pneumoniae)*, *Shigella sonnei* (*S*. *sonnei*), *Mycobacterium tuberculosis* (*M*. *tuberculosis*), and *Clostridium difficile* (*C*. *difficile*). We first conduct a literature review to obtain estimates of *W*, *M*, *L* and *R*_*0*_ for each pathogen and then estimate transmission divergence using simulations. To compare estimates of transmission divergence under different models, we use two approaches described in the literature, namely the *outbreaker* model by Jombart *et al*. [5] and the *phybreak* model by Klinkenberg *et al*. [26] These differ significantly in their model of sequence evolution, with the prior considering a single dominant pathogen strain within each host and the latter modelling the additional complexities of multiples lineages coexisting and coalescing within host. Finally, we illustrate the impact of transmission divergence on our ability to infer transmission trees, using the *outbreaker* and *phybreak* inference algorithms for the R software [27].

## Results

### Transmission divergence

**Fig 1:**
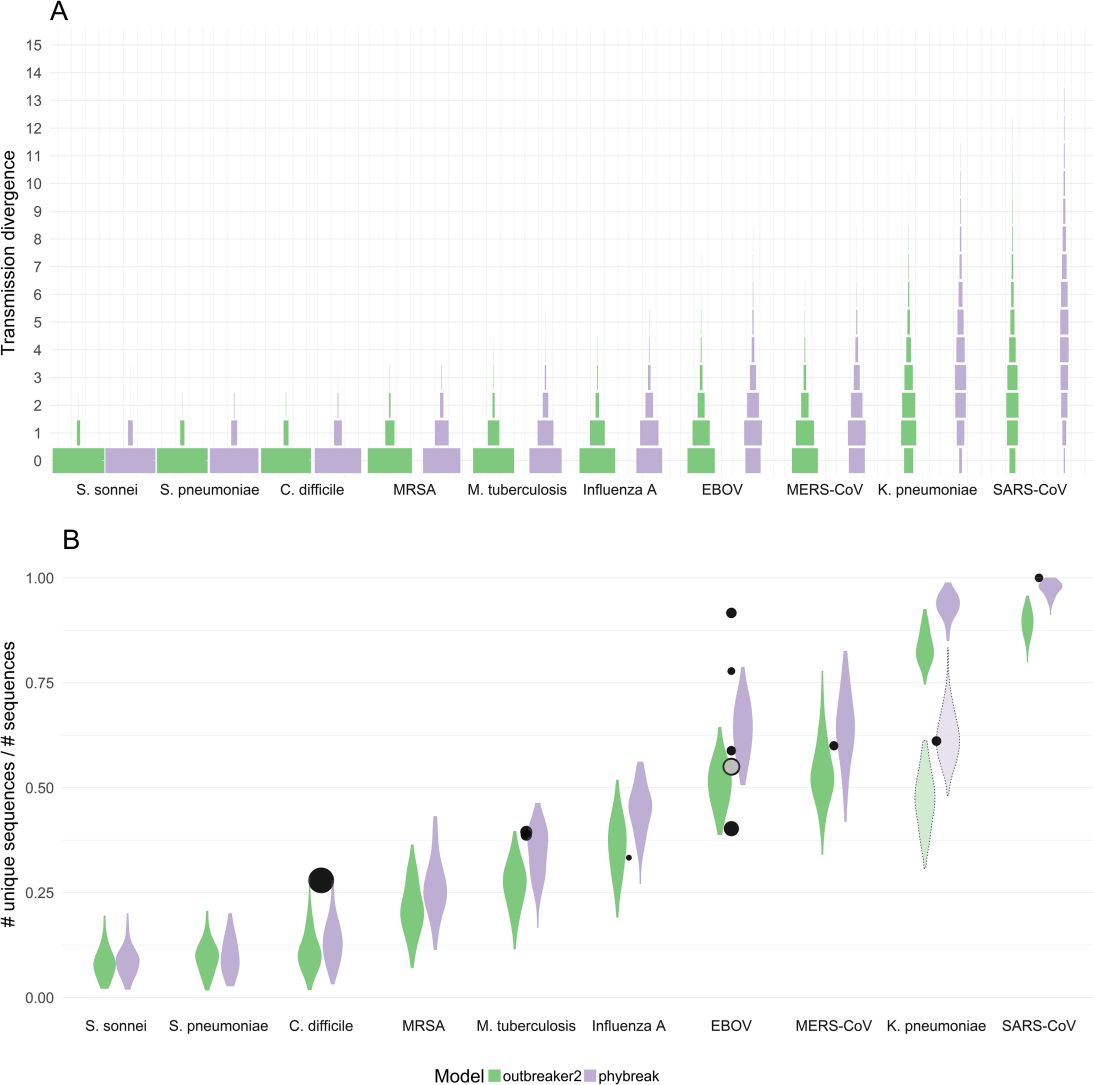
Distributions of simulated transmission divergence values for different pathogens using the *outbreaker* and *phybreak* models. **A)** Transmission divergence is defined as the number of mutations separating pathogen WGS sampled from transmission pairs. Horizontal bars indicate the proportion of transmission pairs separated by that number of mutations, across 100 outbreak simulations per pathogen. Outbreaks were simulated using both the *outbreaker* and *phybreak* models. **B)** For each simulated outbreak, we calculated the proportion of sequences that were unique. Black circles represent empirical observations of the proportion of unique sequences for a given outbreak (S6 Table), scaled by the size of the outbreak. The grey circle in the EBOV column represents the weighted mean across the four outbreaks. The violin plots with the dotted outlines in the *K*. *pneumoniae* column were generated using the empirical serial interval of 25.8 days observed over the course of the outbreak described by Snitkin *et al*. [106], which differs significantly from the value of 62.7 days in our literature review.

We conducted a literature review to obtain, for each pathogen, estimates of *W* (S1 Table), *M* (S2 Table), *L* (S3 Table) and *R*_0_ (S4 Table), and used these to simulate outbreaks under the *outbreaker* and *phybreak* models (Table 1). Simulated outbreaks varied in size from 30 to 99 infected individuals, with a median size of 63 and 62 cases for *outbreaker* and *phybreak* simulations, respectively. To describe the distribution of transmission divergence values for each pathogen, we calculated the number of mutations separating individual transmission pairs (Fig 1A, S5 Table). As expected from the mutational models of *outbreaker* and *phybreak*, transmission divergence appears to follow a mixed Poisson distribution, with the mixing distribution of the Poisson rate determined by variation in the generation-, sampling- and coalescent times.

**Table 1:** Epidemiological and genomic parameters for ten major outbreak causing pathogens.

Pathogen	Generation time (SD) (in days)	Mutation rate (per site per day)	Genome length (base pairs)	Basic reproduction number R_0_	References
***EBOV***	14.4 (8.9)	0.31 x 10^−5^	18958	1.8	[28–34]
***MERS-CoV***	10.7 (6.0)	0.25 x 10^−5^	30115	1.2	[35–40]
***SARS-CoV***	8.7 (3.6)	1.14 x 10^−5^	29714	2.7	[41–49]
***Influenza A (H1N1)***	3.0 (1.5)	1.19 x 10^−5^	13155	1.5	[50–56]
***MRSA***	15.6 (10.0)	5.21 x 10^−9^	2842618	1.3	[57–68]
***K. pneumoniae***	62.7 (24.0)	6.30 x 10^−9^	5305677	2.0	[69–78]
***S. pneumoniae***	6.6 (1.8)	5.44 x 10^−9^	2126652	1.4	[79–88]
***M. tuberculosis***	324.4 (384.5)	0.24 x 10^−9^	4411621	1.8	[11,12,18,89–94]
***S. sonnei***	8.5 (3.0)	1.64 x 10^−9^	4825265	1.1	[95–100]
***C. difficile***	28.4 (14.9)	0.88 x 10^−9^	4290252	1.5	[23,101–105]

Transmission divergence simulated under the two models differed significantly, with *phybreak* consistently estimating higher values than *outbreaker* (S5 Table). This discrepancy ranged from 1.56 times higher on average for *S*. *pneumoniae* to 1.84 times higher for *M*. *tuberculosis*, with significantly longer tailed distributions especially for *K*. *pneumoniae* and SARS-CoV. On the other hand, *outbreaker* and *phybreak* agreed on the relative amount of transmission divergence between pathogens, both assigning larger mean transmission divergence to viral pathogens than bacterial pathogens, with the exception of *K*. *pneumoniae*.

Notable across both models was the fact that transmission divergence was generally low. Pathogens such as *S*. *sonnei*, *S*. *pneumoniae* and *C*. *difficile* were essentially never separated by more than one mutation even when accounting for within-host diversity, suggesting that little to no genetic diversity is to be expected over the course of such outbreaks. Even rapidly mutating viral pathogens such as EBOV and MERS were generally separated by no more than five mutations under both models, and in the absence of significant within-host diversity the most common number of mutations separating such transmission pairs was indeed zero. In fact, *outbreaker* estimated a mean value below one for eight of the ten pathogens considered. In contrast, two pathogens that demonstrated significantly higher transmission divergence were *K*. *pneumoniae* and SARS-CoV, which accumulated as many as 15 mutations between individual transmission pairs and were rarely separated by fewer than two.

We also quantified genetic diversity by the number unique of sequences as a proportion of total sequences (Fig 1B, S5 Table). This value is closely related to the zero term in the transmission divergence distribution, however notable observations include that over 90% of sequences in *S*. *sonnei* and *S*. *pneumoniae* outbreaks were identical under both models of sequence evolution, and on average 30% to 50% of sequences in MERS-CoV and EBOV outbreaks were identical depending on the model of within-host diversity. Few genetically identical cases were observed in SARS-CoV and *K*. *pneumoniae* outbreaks.

### Comparison with empirical results

As the proportion of unique sequences in an outbreak can be determined without knowledge of the transmission tree, we used this metric to compare our predictions with empirical estimates from studies collecting WGS in an outbreak setting (Fig 1B, S6 Table). The proportion of unique sequences observed in *M*. *tuberculosis*, Influenza A, MERS-CoV and SARS-CoV outbreaks were well predicted by one or both evolutionary models. The *phybreak* model better predicted the observed genetic diversity for both *M*. *tuberculosis* outbreaks and the SARS-CoV outbreak, the latter of which fell outside the prediction interval of the *outbreaker* model, whereas the diversity observed in the Influenza A and MERS-CoV outbreaks was similarly supported by both models.

The mean proportion of unique sequences observed across four EBOV outbreaks was also in good agreement with our simulations, with slightly greater support by the *outbreaker* model. However, diversity between these outbreaks was more variable than expected, ranging from 0.40 to 0.92, with one value falling outside both prediction intervals and two values only expected under either the *phybreak* or *outbreaker* model. Furthermore, though the genetic diversity observed across 333 cases of *C*. *difficile* infection in Oxfordshire, UK [17] fell just within the prediction interval of our simulations, this result was unlikely under both evolutionary models, especially given the large sample size of the study.

**Fig 2:**
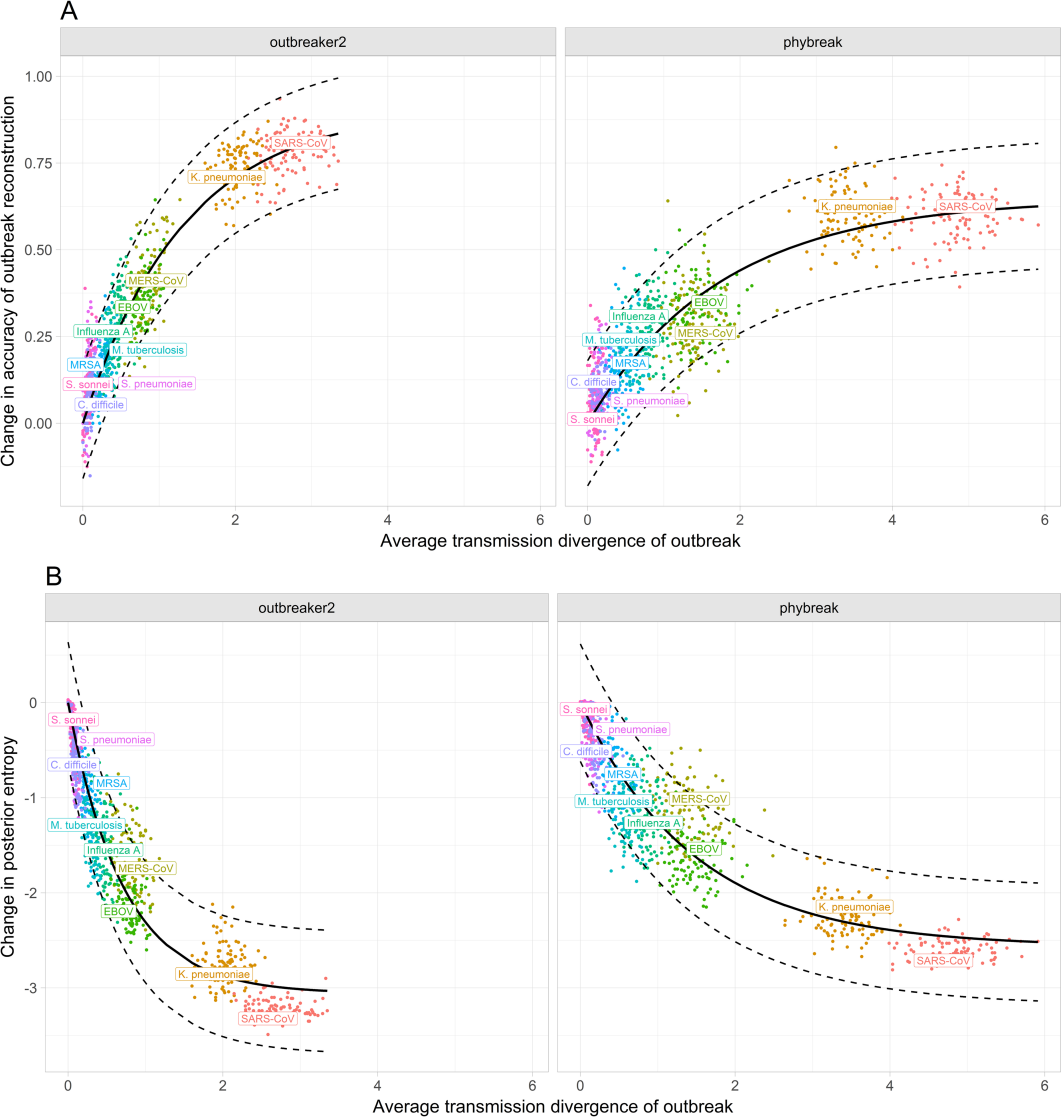
Impact of transmission divergence on outbreak reconstruction. Transmission divergence is defined as the number of mutations separating pathogen WGS sampled from transmission pairs. **A) Change in accuracy of outbreak reconstruction.** Accuracy of outbreak reconstruction is defined as the proportion of correctly assigned ancestries in the consensus transmission tree, itself defined as the tree with the most frequent posterior infector for each infectee. Coloured points represent individual simulated outbreaks. The solid black line represents the fitted relationship of the form *i*—i*exp(-a*K), where *K* is the transmission divergence and *a* and *i* the fitting variables. Dotted black lines represent the corresponding 95% prediction interval. **B) Change in posterior entropy.** Posterior entropy is related to the number of plausible posterior infectors for a given case. Lower average entropy indicates greater statistical confidence in the proposed transmission tree. The solid black line represents the fitted relationship of the form i*exp(-a*K)—*i*, where *K* is the transmission divergence and *a* and *i* the fitting variables.

The greatest disagreement with our predictions was observed for a *K*. *pneumoniae* outbreak described by Snitkin *et al*. [106], for which 7 out of 18 WGS were identical, while our simulations predicted nearly all cases to be genetically distinguishable. However, the average serial interval over this outbreak was only 25.8 days (S6 Table), which was unusually short compared to the average value of 62.7 days from our literature review. When repeating our simulations using the realised serial interval, the observed genetic diversity was well predicted by the *phybreak* model (Fig 2B).

### Impact on outbreak reconstruction

To quantify how these results affect the inference of transmission trees, we analysed the simulated outbreaks using the *outbreaker* and *phybreak* inference algorithms, applying the same models used for outbreak simulation in their reconstruction. We reconstructed each outbreak with and without WGS data, and quantified the accuracy in outbreak reconstruction, as well as the statistical confidence in ancestry assignments using the posterior entropy (S1 Fig, S2 Fig). To describe the informativeness of the genetic data alone, and minimise confounding epidemiological differences between pathogens (e.g. temporal data will be more informative with lower R_0_, as there exist longer chains with a greater temporal signal), we calculated the absolute change in accuracy of outbreak reconstruction upon incorporating WGS data and related this to the mean transmission divergence of the outbreak. As the accuracy of reconstruction in the absence of WGS data was very low (S1 Fig), there was similar, considerable scope for improvements in accuracy for all pathogens.

Unsurprisingly, higher average transmission divergence across an outbreak led to greater improvements in the accuracy of outbreak reconstruction of both *outbreaker* and *phybreak* simulations (Fig 2A). However, the nature of this relationship differed between the two models. Using *outbreaker*, a sharp contrast was observed between outbreaks with low mean transmission divergence (*C*. *difficile*, *S*. *pneumoniae*), for which WGS provided essentially no additional information, and outbreaks exhibiting the largest mean transmission divergence (*K*. *pneumoniae*, SARS-CoV), for which WGS improved nearly every incorrect ancestry assignment. The effect of mean transmission divergence on the accuracy of outbreak reconstruction was strongly nonlinear, with the greatest improvement in accuracy obtained between 0 and 1 mutations on average between transmission pairs.

Under the *phybreak* model this relationship was less pronounced, with increases in mean transmission divergence resulting in markedly lower improvements in accuracy (Fig 2A). This was most evident in SARS-CoV and *K*. *pneumoniae* outbreaks, for which improvements in accuracy were lower than in the *outbreaker* simulations even though the average transmission divergence was nearly two times higher, with a significant number of ancestries remaining incorrectly assigned (S1 Fig). At high values, mean transmission divergence was also poorly predictive of increases in accuracy, which were identical between SARS-CoV and *K*. *pneumoniae* in spite of significantly different average transmission divergence (4.83 and 3.40, respectively).

A similar trend was observed when considering the statistical confidence in ancestry assignments (Fig 2B). Under the *outbreaker* model, higher average transmission divergence strongly reduced posterior entropy, resulting in essentially complete support for a single transmission tree at high values. In contrast, the consideration of within-host diversity by the *phybreak* model left outstanding uncertainty around ancestry assignments even when transmission pairs were resolved by a large number of mutations (S2 Fig).

**Fig 3:**
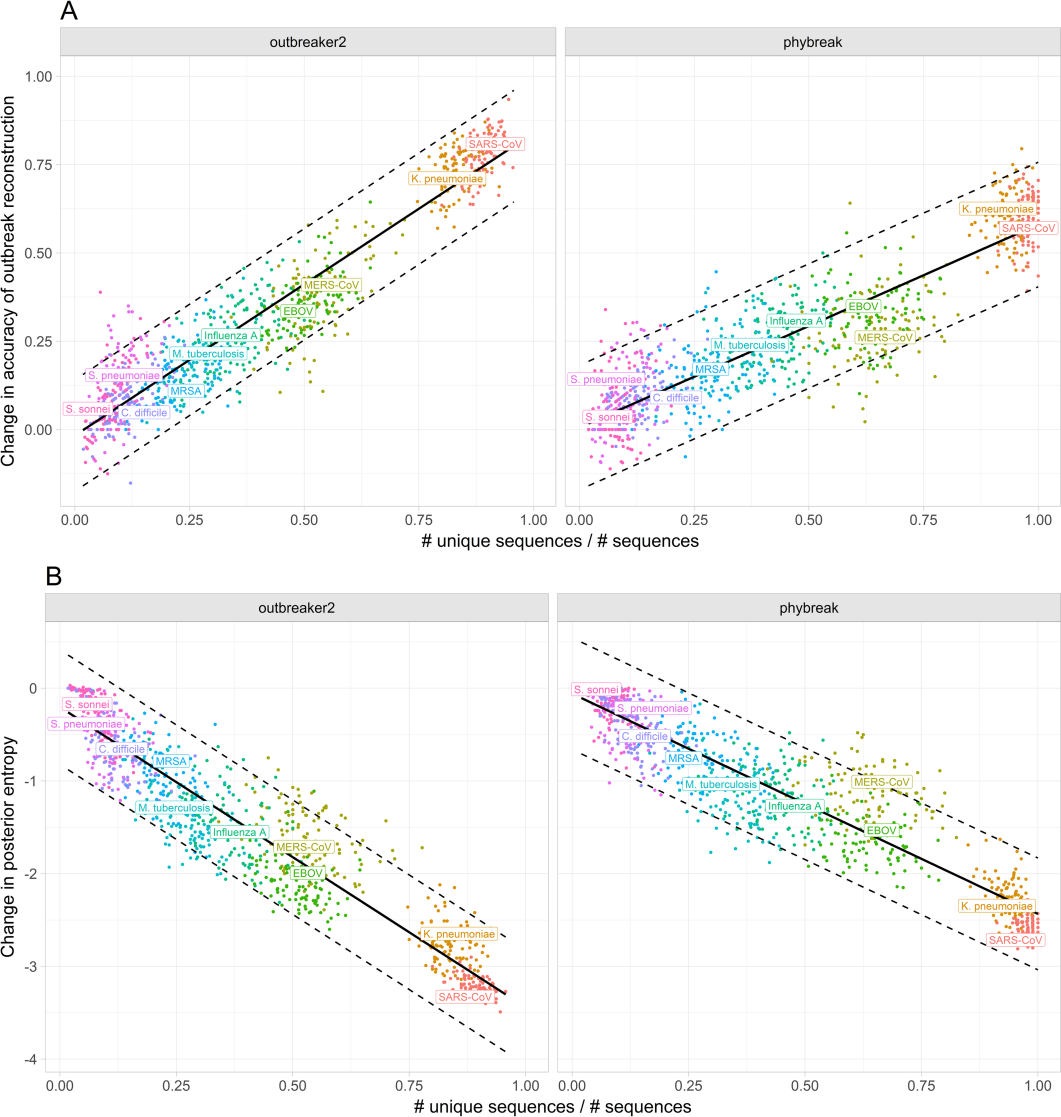
Impact of the proportion of unique sequences on outbreak reconstruction. **A) Change in accuracy of outbreak reconstruction.** Accuracy of outbreak reconstruction is defined as the proportion of correctly assigned ancestries in the consensus transmission tree, itself defined as the tree with the most frequent posterior infector for each infectee. Coloured points represent individual simulated outbreaks. The solid black line represents the fitted linear model, the dotted black lines the 95% prediction interval. **B) Change in posterior entropy.** Posterior entropy is related to the number of plausible posterior infectors for a given case. Lower average entropy indicates greater statistical confidence in the proposed transmission tree. The solid black line represents the fitted linear model, the dotted black lines the 95% prediction interval.

We also related the informativeness of WGS data to the proportion of sequences that were unique in an outbreak, and found a nearly linear relationship for both *outbreaker* and *phybreak* reconstructions (Fig 3A). Once again, the slope of this relationship was significantly steeper in the *outbreaker* model, to the extent that the proportion of unique sequences was a near perfect predictor of the informativeness of WGS data, and outbreaks essentially perfectly reconstructed if all sequences were genetically distinct. This was not the case with the *phybreak* reconstructions, which assigned incorrect ancestries even when all sequences were unique. However, the proportion of unique cases was still a good predictor of the increase in accuracy of outbreak reconstruction, and successfully identified *K*. *pneumoniae* and SARS-CoV as having similarly informative WGS, where mean transmission divergence as a metric had placed them far apart.

The change in posterior entropy was also linearly correlated with the proportion of unique ancestries (Fig 3B), with an outbreak of genetically distinct cases sufficient for *outbreaker* to converge on a single posterior transmission tree. In contrast, there remained considerable uncertainty around *phybreak* reconstructions even in a fully genetically resolved outbreak.

## Discussion

This paper has introduced the concept of ‘transmission divergence’ as a measure of the informativeness of WGS data for reconstructing transmission chains during an infectious disease outbreak. We estimated transmission divergence for ten major outbreak causing pathogens with a simulated based approach, using two distinct models of sequence evolution for comparison. We then demonstrated how the mean transmission divergence across an outbreak affects our ability to infer transmission histories.

Though average transmission divergence varied significantly amongst the diseases studied, it was generally very low, with a modal value of zero for most pathogens under both evolutionary models. Our results suggest that a large fraction or even a majority of cases will be genetically indistinguishable in many epidemic scenarios, including outbreaks of rapidly evolving RNA viruses such as EBOV and MERS.

These results were generally well supported by empirical observations of genetic diversity. Our simulations accurately predicted *C*. *difficile*, Influenza A and *M*. *tuberculosis* cases to be genetically identical a majority of the time, EBOV and MERS-CoV cases to display greater diversity yet still be frequently identical, and SARS-CoV cases to be largely genetically distinct. Though the proportion of unique sequences observed across 333 *C*. *difficile* cases was unexpectedly high, this metric assumes that each case is related to another case by a direct transmission event. This appears unrealistic when considering that 120 patients (36%) had no recorded epidemiological contact with their genetically related case [17]. A number of these pairs were probably separated by unobserved transmission events, for example by asymptomatic carriers, increasing the observed genetic diversity between supposed transmission pairs. The true proportion of unique sequences likely agrees better with our model predictions. Furthermore, though our original estimates for *K*. *pneumoniae* disagreed with empirical observations, this discrepancy was largely due to an unusually short serial interval compared to previously reported values in the literature. Once accounted for, the reported diversity agreed with our predictions.

Therefore, even though predicting the specific number of unique sequences observed in an outbreak is challenging due to confounding factors such as unobserved cases and stochastic variations in epidemiological context, our predictions using two fairly simple models of sequence evolution largely agreed with the data. This suggests that our results represent broadly useful predictors of the extent to which cases in a transmission cluster are genetically resolvable. The wider conclusion that a significant proportion of cases are expected to be genetically identical for a number of different pathogens is certainly well supported.

It is tempting to suggest that the data from *M*. *tuberculosis* and SARS-CoV outbreaks lend greater support to the *phybreak* model, thereby indicating significant levels of within-host genetic diversity among these pathogens. While this explanation is feasible, the significant variation in unique sequences across four EBOV outbreaks demonstrates the sensitivity of such individual observations to stochastic effects. In the absence of greater amounts of empirical data, any such conclusions are only weakly supported.

The limited genetic diversity as predicted by our simulations had a considerable impact on our ability to reconstruct outbreaks, and clearly identified transmission divergence as a limiting factor in the utility of WGS data for many pathogens in an outbreak setting. These informational limitations were further compounded by within-host genetic diversity, which significantly reduced our ability to reconstruct outbreaks even when mean transmission divergence was high, in agreement with previous studies [11,107]. Combined, these results demonstrate that WGS data will often be insufficient to fully resolve transmission chains, and reveal the need to incorporate other sources of information into transmission inference frameworks. Promising avenues include an analysis of deep sequencing data as an alternative to WGS, which may reveal additional within-host variation informative of likely transmission events, as well as a methodological approach to inferring transmission routes from contact data.

Our results do not imply that WGS is of no use for inferring transmission routes as a whole. For example, Didelot *et al*. used *C*. *difficile* WGS to identify distinct transmission chains caused by separate introductions to the same ward, vastly reducing the number of plausible transmission links given only epidemiological data [23]. However, samples within the transmission chains were genetically identical and remained unresolved in the absence of additional data. Low transmission divergence therefore represents a hard limit to the resolution of various reconstruction methods using WGS as primary source of information, regardless of the underlying genetic model. This may especially impact approaches relying on previously constructed phylogenetic trees [12,14], which are known to skew infection time estimates in the presence of multiple genetically identical sequences, as described by Hall *et al*. [15]

We also showed that greater transmission divergence generally improved the inference of transmission histories, however only to an extent. Beyond the first discriminatory mutation, diversity between transmission pairs seemed to provide limited additional information, as demonstrated by the fact that the proportion of genetically unique sequences, rather than the average transmission divergence, best predicted the informativeness of WGS data. Though this relationship was weaker in the *phybreak* model, as within-host diversity increases the number of plausible transmission trees even when all cases are genetically distinct, this linear relationship held across both models.

It is important to note that other epidemiological factors beyond genetic diversity will impact the accuracy of outbreak reconstruction, such as R_0_, heterogeneities in infectiousness, the generation time distribution and the sampling time distribution. To account for these effects, our study focused on the improvement in the accuracy of reconstructed transmission chains, compared to a baseline without WGS data while keeping these other factors constant. Importantly, in spite of considerable variation in R_0_ and generation time distributions (Table 1), the robustness of the correlations presented in Figs 2 and 3 suggests that our measures of genetic diversity have captured a central determinant of the utility of WGS data in reconstructing outbreaks.

This study made several assumptions which might be relaxed in further work. Firstly, we assumed that the sampling time distribution is equivalent to the generation time distribution. While this assumption was largely driven by the lack of available data on sampling delay distributions, this could result in biases. For instance, our approach would underestimate transmission divergence in the presence of systematic and substantial lags between transmission times and sampling (S3 Fig). It is worth noting, however, that additional mutations accumulating in a lineage after onward transmission has ceased would increase the overall genetic distance from this lineage to all other isolates equally, without providing additional information about the underlying epidemiological relationships between hosts. Therefore, it is unclear how this additional diversity would translate in terms of improving outbreak reconstruction, and we believe the approach used in this study should capture the diversity informative of the transmission network.

Secondly, outbreaks were simulated and reconstructed under idealised scenarios, in that all cases were observed, WGS were available for all cases, and the same parameter values used for simulation and inference. Most importantly, we assumed error-free sequencing. In reality, when considering that transmission divergence is generally on the order of single mutations, individual sequencing errors can heavily bias the topology of the inferred transmission tree. Our estimates of the informativeness of WGS in inferring individual transmission links are therefore likely to be optimistic.

Finally, both the *outbreaker* and the *phybreak* model assumed a complete bottleneck at transmission, with a single strain being transmitted. Allowing for an incomplete bottleneck greatly increase the complexity of the problem, as two strains in a given host may have diverged several infectious generations ago and passed through multiple bottleneck together. This issue also opens up a number of questions about optimal sampling and sequencing strategies, the exact magnitude of the genetic diversity bottleneck at transmission, and the more fundamental mechanisms permitting the coexistence of multiple strains within a host. Further work should be dedicated to investigating the impact of these issues on the use of WGS for outbreak reconstruction [107,108].

The advent of WGS data has initiated a revolution in modern infectious disease epidemiology, shedding new light into disease dynamics and evolution at a variety of scales [109,110]. At a local scale, these data have opened up exciting perspectives for improving our understanding of the person-to-person transmission process [5,6,13,20]. This work suggests that, while useful, the analysis of WGS alone will struggle to reconstruct transmission trees accurately for a large number of pathogens, in particular bacterial ones. Integrating other types of outbreak data, such as locations of patients, community structure, or contact tracing data, therefore represents a promising alternative strategy for outbreak reconstruction.

## Materials and methods

### Epidemiological and genomic parameters

We conducted a literature review using OvidEmbase to attain values for the generation time distribution, basic reproduction number, mutation rate and genome length of the pathogens under consideration. The searches were performed between 1st June 2015 and 3rd March 2017 and limited to publications in English. Common name variants for each pathogen were included in the searches as follows:

1. Ebola OR Ebola virus
2. MERS or MERS-CoV OR Middle East respiratory syndrome
3. SARS OR SARS-CoV OR Severe acute respiratory syndrome
4. (Influenza OR flu) AND (H1N1 OR A(H1N1) OR pandemic)
5. *Staphylococcus aureus* OR MRSA OR Methicillin resistant *Staphylococcus aureus*
6. Klebsiella pneumoniae
7. Streptococcus pneumoniae
8. Mycobacterium tuberculosis
9. Shigella sonnei
10. Clostridium difficile

For the generation time distribution, we used the search terms ‘generation time OR serial interval OR generation interval’. Estimates of the serial interval were used as a proxy for the generation time. We summarised the mean and standard deviation of these distributions by calculating the arithmetic mean weighted by the sample size of the study. We then generated discretized gamma distributions of the generation time distribution, using the function *DiscrSI* from the R package *EpiEstim* [111].

Studies describing the mutation rate were identified using the search terms ‘mutation rate OR substitution rate OR spontaneous mutation’. The arithmetic mean was calculated to summarise findings (Table 1). A discrepancy of two orders of magnitude between mutation rate estimates for *Clostridium difficile* was resolved by choosing the short-term molecular clock estimate, derived from serial pairs of isolates in a hospital outbreak [23], over a long-term estimate using historical phylogenetic analysis [112]. Mutation rates were converted to units of mutations per site per day.

Core genome length estimates were retrieved from complete genome assemblies in the GenBank repository [113], and the rounded arithmetic mean used as a summary value.

Studies estimating *R*_0_ were identified using the search terms: ‘basic reproduction number OR basic reproductive number’. Only studies explicitly inferring the *basic* reproduction number, defined as the expected number of secondary infections caused by an index case in a wholly susceptible population, were selected. The arithmetic mean was used as a summary value.

### Estimating transmission divergence from simulated outbreaks

We define the transmission divergence *K* as the number of mutations separating pathogen WGS sampled from transmission pairs. We estimated the distribution of values for *K* by simulating transmission events alongside sequence evolution under two different models. The first is the *outbreaker* model, described in full by Jombart *et al*. [5] Briefly, the infectiousness of a case at a given time since infection is described by the generation time distribution *W* scaled by the basic reproduction number *R*_*0*_. The time of sampling is drawn from the sampling time distribution *S*. Mutations accumulate in the time between infection of a primary and secondary case, at a daily rate given by the product of the genome length *L* and the mutation rate *M*. Within-host pathogen diversity is considered negligible, such that the same strain is both onwardly transmitted and sampled, and the bottleneck at transmission is assumed complete.

The *phybreak* model is described by Klinkenberg *et al*. [26], and differs from the *outbreaker* model primarily in its model of sequence evolution. Instead of modelling mutations as independent events between individual transmission pairs, *phybreak* accounts for patterns of shared evolution and within-host diversity by simulating phylogenetic ‘mini-trees’ within each case, which are combined according to the transmission tree. The coalescent events within-host are simulated under a linearly growing pathogen population size, assuming a complete bottleneck at transmission. Mutations accumulate along the branches as a Poisson process with a mean value of the mutation rate *M*. As with *outbreaker*, infection times and sampling times are drawn from the generation time distribution *W* and sampling time distribution *S*, respectively. The number of contacts is Poisson distributed with a mean of *R*_*0*_, resulting in transmission if these occurred with previously uninfected cases.

### Simulation settings

For both *outbreaker* and *phybreak*, we simulated outbreaks with 100 susceptible hosts and a single initial infection using parameter values for *M*, *W*, *L* and *R*_*0*_ obtained by literature review. The sampling time distribution was assumed to be the equivalent to the generation time distribution, and external imports of infection were not considered. Within-host evolution was modelled in *phybreak* with an effective pathogen population size increasing at a daily rate of 1. Simulations were run for 500 days or until no more infectious individuals remained, except *M*. *tuberculosis* simulations which were run for 500 weeks due to the longer generation time. For each pathogen, we generated 100 *outbreaker* simulations and 100 *phybreak simulations* with a minimum size of 30 infected individuals. The distributions of transmission divergence values were extracted by determining the number of mutations separating each transmission pair.

### Outbreak reconstruction

We reconstructed *outbreaker* and *phybreak* simulations using the transmission tree inference algorithms in the *outbreaker2* and *phybreak* packages, respectively, which implement the same models used for simulation described above. The generation time and sampling time distributions used for simulations were also used for inference, and the assumed rate of within-host population growth in *phybreak* fixed at the simulated value. *outbreaker* MCMC chains were run for 100,000 iterations with a thinning frequency of 1/200, and *phybreak* MCMC chains for 10,000 iterations with a thinning frequency of 1/20. The burn-in period for both analyses was 1,000 iterations. To assess the improvement in transmission tree reconstruction due to genetic data alone, two types of analyses were performed for each simulated dataset, the first one using only sampling times, and the second one using both sampling times and WGS data. As the phybreak algorithm requires WGS data to be provided, all cases were assigned identical genomes to imitate the absence of genetic information.

For each simulation, we quantified the accuracy of outbreak reconstruction as the proportion of correctly inferred ancestries in the consensus transmission tree, defined as the tree with the modal posterior infector for each sampled case. Cycles were resolved using Edmond’s algorithm [114]. The change in accuracy was defined as the absolute difference in accuracy upon inclusion of WGS data.

To quantify the statistical confidence in ancestry assignments contained in the posterior distribution, we calculated the entropy of posterior ancestries for each case [115]. Given *K* ancestors of frequency *f*_*K*_ (*k* = 1, …,*K*), the entropy is defined as:

$$-\sum_{k=1}^{K}f_{k}log\left(f_{k}\right)$$ (1)

An entropy value of 0 therefore indicates complete posterior support for a given ancestry, with higher values indicating a larger number of plausible transmission scenarios.

## Supporting information

S1 Fig Accuracy of outbreak reconstruction with and without WGS. 100 outbreaks were simulated and reconstructed for each pathogen, using both the *outbreaker* and *phybreak* model. Accuracy of outbreak reconstruction is defined as the proportion of correctly assigned ancestries in the consensus transmission tree, itself defined as the tree with the most frequent posterior infector for each infectee.
(TIF)

S2 Fig Posterior entropy with and without WGS. 100 outbreaks were simulated and reconstructed for each pathogen, using both the *outbreaker* and *phybreak* model. Posterior entropy is related to the number of plausible posterior infectors for a given case, with lower average entropy indicating greater statistical confidence in the proposed transmission tree.
(TIF)

S3 Fig Quantifying the time for mutations to accumulate between pathogen genomes sampled from a transmission pair under the *outbreaker* model. Individual *i* infects individual *j*. Infection and sampling times are indicated by circles and diamonds, respectively. The generation time *W*_*i*,*j*_ is defined as the intervals between infection of *i* and the secondary case *j*, and is drawn from the distribution W. S_*i*_ denotes the time to sampling of individual *i*, and is drawn from the distribution *S*. The time for discriminatory mutations to occur between pathogen genomes sampled from *i* and *j* is denoted O_i,j_, and is represented by red lines.
**A.** If sampling of *i* occurs after onwards infection:
O_i,j_ = S_i_—W_i,j_ + S_j_
E(O_i,j_) = 2*E(*S*)—E(*W*)
If the difference between the expected generation time and expected time to sampling is negligible:
E(O_i,j_) ≈ E(*W*)
**B.** If sampling of *i* occurs before onwards infection:
O_i,j_ = W_i,j_—S_i_ + S_j_
E(O_i,j_) = E(*W*)
The time for mutations to occur is well approximated by the generation time if the delay between sampling and onwards infection is small. If sampling consistently occurs long after onwards infection, the time for mutations to occur will be underestimated.
(TIF)

S1 Table Generation time distributions. (DOCX)

S2 Table Mutation rates. (DOCX)

S3 Table Genome lengths. (DOCX)

S4 Table Basic reproduction number R0. (DOCX)

S5 Table Transmission divergence and its effect on outbreak reconstruction for different pathogens. (DOCX)

S6 Table Proportion of unique WGS collected in an outbreak setting. (DOCX)
