# Supplementary figures and images for: When are pathogen genome sequences informative of transmission events?
Source: PLoS Pathog. 2018 Feb 8;14(2):e1006885. doi: 10.1371/journal.ppat.1006885 (PMC5821398; doi:10.1371/journal.ppat.1006885)

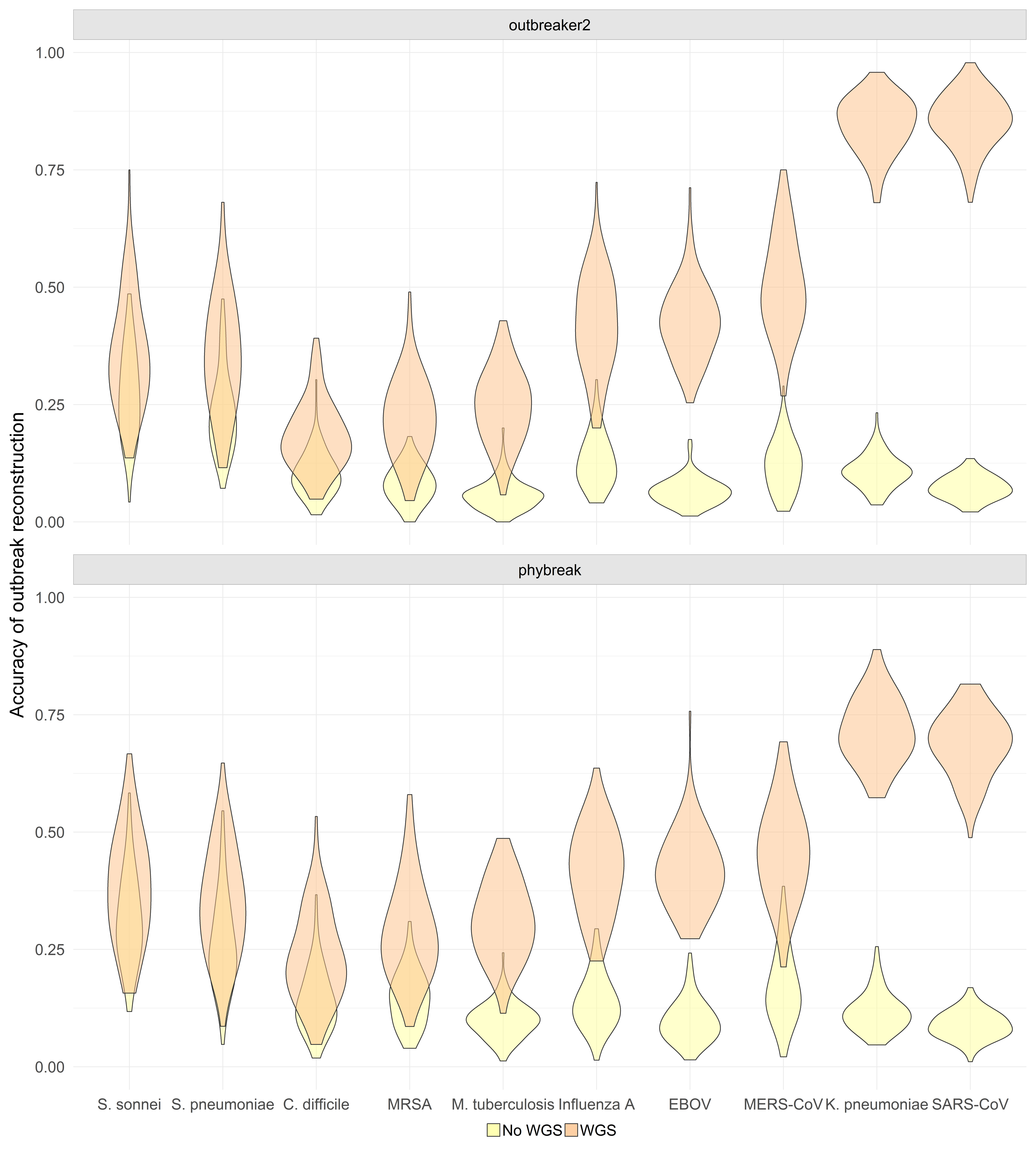

Supplement: S1 Fig — 100 outbreaks were simulated and reconstructed for each pathogen, using both the outbreaker and phybreak model. Accuracy of outbreak reconstruction is defined as the proportion of correctly assigned ancestries in the consensus transmission tree, itself defined as the tree with the most frequent posterior infector for each infectee. (TIF) [file ppat.1006885.s001.tif]

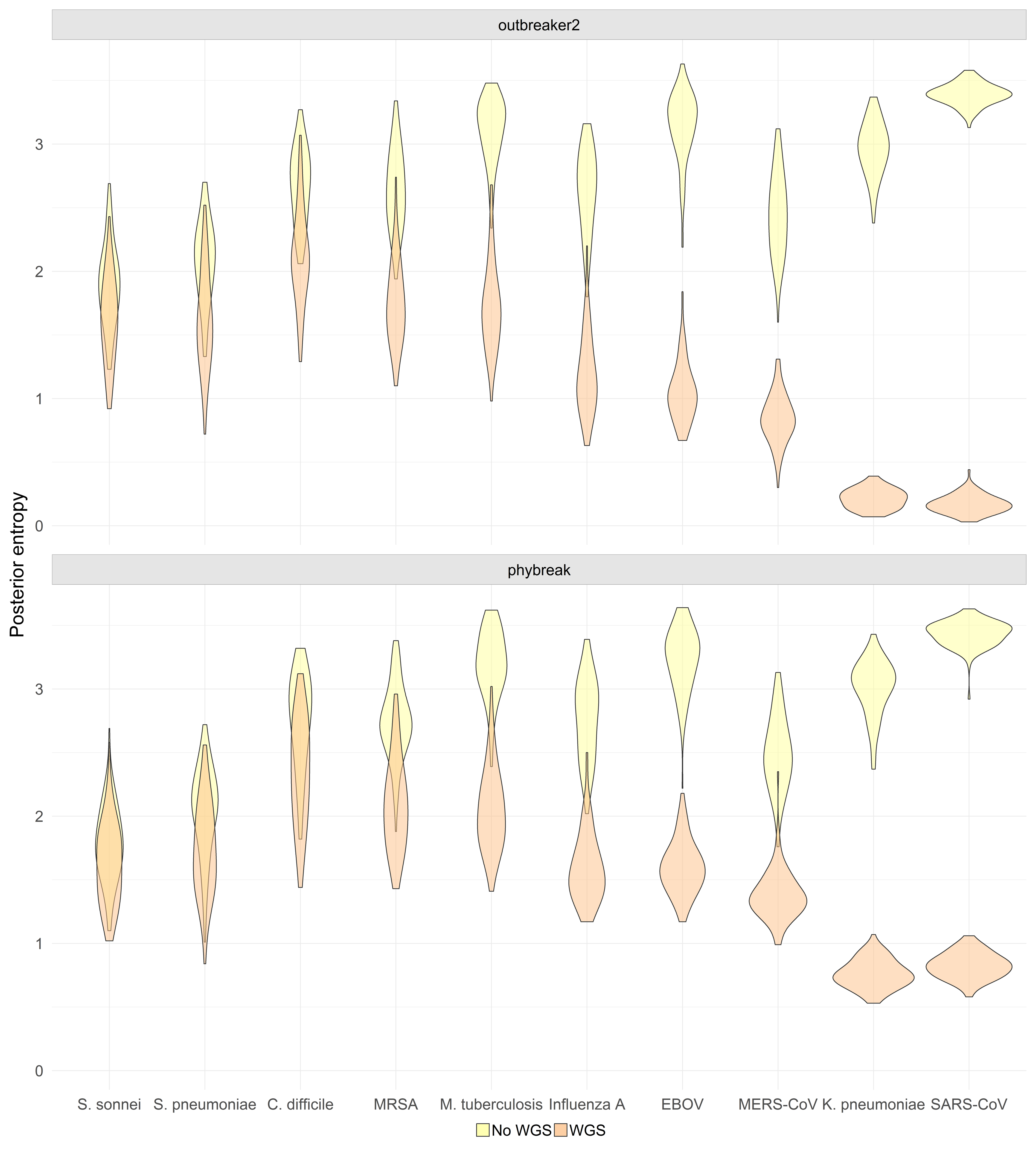

Supplement: S2 Fig — 100 outbreaks were simulated and reconstructed for each pathogen, using both the outbreaker and phybreak model. Posterior entropy is related to the number of plausible posterior infectors for a given case, with lower average entropy indicating greater statistical confidence in the proposed transmission tree. (TIF) [file ppat.1006885.s002.tif]

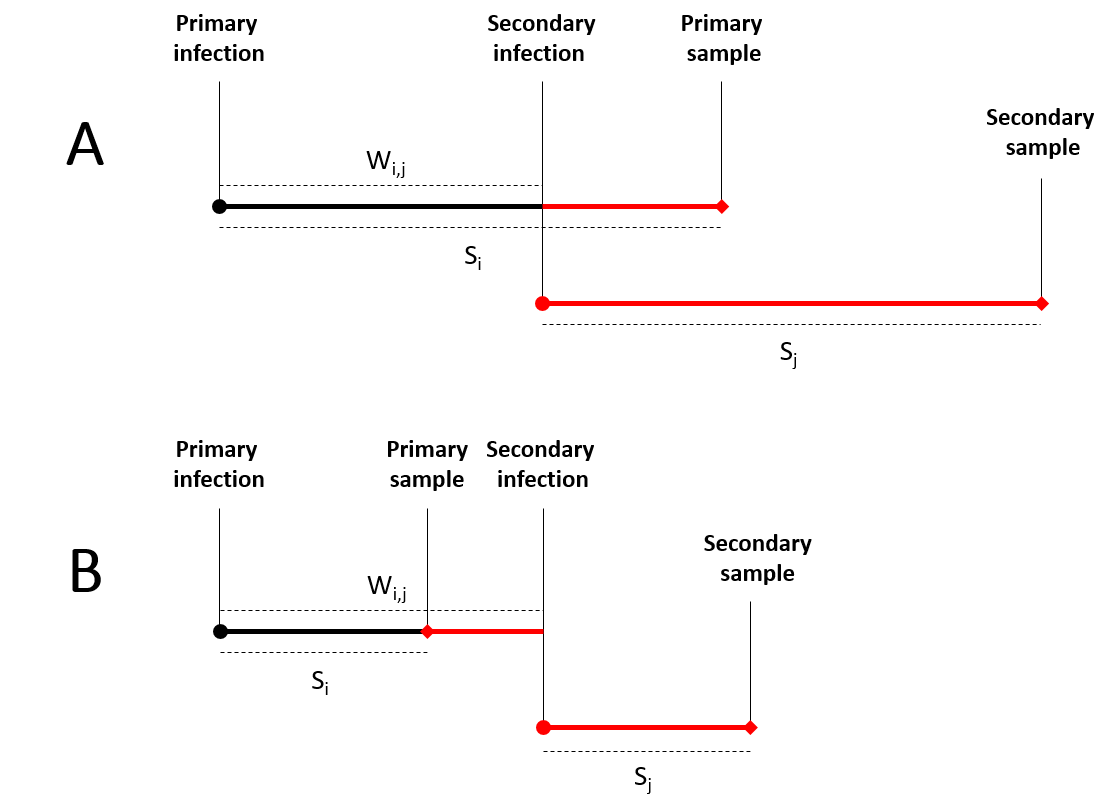

Supplement: S3 Fig — Individual i infects individual j. Infection and sampling times are indicated by circles and diamonds, respectively. The generation time Wi,j is defined as the intervals between infection of i and the secondary case j, and is drawn from the distribution W. Si denotes the time to sampling of individual i, and is drawn from the distribution S. The time for discriminatory mutations to occur between pathogen genomes sampled from i and j is denoted Oi,j, and is represented by red lines. A. If sampling of i occurs after onwards infection: Oi,j = Si—Wi,j + Sj E(Oi,j) = 2*E(S)—E(W) If the difference between the expected generation time and expected time to sampling is negligible: E(Oi,j) ≈ E(W) B. If sampling of i occurs before onwards infection: Oi,j = Wi,j—Si + Sj E(Oi,j) = E(W) The time for mutations to occur is well approximated by the generation time if the delay between sampling and onwards infection is small. If sampling consistently occurs long after onwards infection, the time for mutations to occur will be underestimated. (TIF) [file ppat.1006885.s003.tif]
