# Supplementary material for: When are pathogen genome sequences informative of transmission events?
Source: PLoS Pathog. 2018 Feb 8;14(2):e1006885. doi: 10.1371/journal.ppat.1006885 (PMC5821398; doi:10.1371/journal.ppat.1006885)
Supplement: S1 Table — (DOCX) [file ppat.1006885.s004.docx]

### **S1 Table. Generation time distributions.**

| **Pathogen** | **Study period and location** | **Sample size** | **Mean (SD) [days]** | **Study method** | **Data description** | **First author,year [reference]** |
| --- | --- | --- | --- | --- | --- | --- |
| **EBOV** | September 2014  Guinea, Liberia,  Sierra Leone | 92 | 15.3 (9.3) | Mean serial interval from fitted gamma distribution | 2014-2015 Outbreak.    Symptom onset in index case and in person infected by index case in known or suspected transmission chains. Sample size refers to the numbers of epidemiologically linked pairs. | WHO ERT, 2014  [[28]](https://paperpile.com/c/YpND2q/ilQz) |
|  | November 2014    Guinea, Liberia, Sierra Leone | 305 | 14.2 (9.6) | Mean serial interval from fitted gamma distribution | 2014-2015 Outbreak    Symptom onset in index case and in person infected by index case. Sample size refers to the numbers of epidemiologically linked pairs. | WHO ERT, 2015 [[29]](https://paperpile.com/c/YpND2q/jVpW) |
|  | October 2014    Guinea | 152 | 14.2 (7.1) | Mean serial interval from fitted gamma distribution to interval censored data | 2014-2015 Outbreak    Earliest symptom onset dates for index case and other cases. | Faye, 2015 [[30]](https://paperpile.com/c/YpND2q/FXSB) |
| **MERS-CoV** | August 2013    France, Jordan, Saudi Arabia, UK | 53 | 10.7 (6.3) | Mean serial interval from fitted gamma distribution | 2012-2013 outbreak.    Earliest symptom onset dates for index case and other cases | Cauchemez, 2014 [[35]](https://paperpile.com/c/YpND2q/DbA3R) |
|  | April 2013    Saudi Arabia | 23 | 9.0 (5.1) | Mean serial interval obtained using quantiles of fitted log normal distribution using R | 2012-2013 outbreak, hospital cases from March 1^st^ to April 19^th^ 2013    Onset date for index and other cases >48 hours pyrexia or 1^st^ day of respiratory symptoms | Assiri, 2013 [[36]](https://paperpile.com/c/YpND2q/3ZsBM) |
|  | November 2012    Saudi Arabia | 4 | 20 (7.8) | Mean empirical serial interval obtained from contact tracing and symptom onset data. | 2012-2013 outbreak, family cluster. Pyrexia onset dates in index case, secondary cases and tertiary case. Index case and secondary cases confirmed, tertiary case probable. | Memish, 2013 [[37]](https://paperpile.com/c/YpND2q/rm1p8) |
| **SARS-CoV** | October 2003    Singapore | 205 | 8.4 (3.8) | Mean of serial interval distribution from fitted Weibull distribution | 2003 outbreak    Symptom onset dates for index case and secondary cases | Lipsitch, 2003 [[41]](https://paperpile.com/c/YpND2q/obgdY) |
|  | March 2003    Vietnam | 28 | 10.1 (1.8) | Mean of empirical serial intervals obtained from symptom onset dates in hospitalised patients. | 2003 hospital outbreak, cases in Northern Vietnam    Symptom onset dates for index case and secondary cases | Reynolds, 2006 [[42]](https://paperpile.com/c/YpND2q/cMTyG) |
|  | February - April 2003    Canada | 8 | 10.3 (4.4) | Mean of empirical serial intervals of cases with symptom onset data from reported transmission chain. | 2003 hospital outbreak    Symptom onset dates for index case, secondary cases and tertiary cases | Varia, 2003 [[43]](https://paperpile.com/c/YpND2q/r7VlS) |
| **Influenza A (H1N1)** | April 2008 – February 2011    Thailand | 125 | 3.3 (1.7) | Mean of serial intervals from fitted lognormal distribution | 2009 pandemic in Thailand    Pyrexia onset dates for index case and other cases | Levy, 2013 [[50]](https://paperpile.com/c/YpND2q/7HSW3) |
|  | June 2009    Netherlands | 32 | 2.7 (1.1) | Mean of empirical serial intervals | 2009 pandemic in Netherlands    Earliest symptom onset dates for index case and other cases | Hahne, 2009 [[51]](https://paperpile.com/c/YpND2q/k48Ge) |
|  | May 2009    USA | 78 | 2.6 (1.3) | Probabilistic modelling accounting for out of household transmission and tertiary cases (12) | 2009 pandemic in USA    Index case: probable/confirmed H1N1 reported to CDC. Other cases: acute respiratory illness in household contacts (37) | Cauchemez, 2009 [[52]](https://paperpile.com/c/YpND2q/6anSO) |
| **MRSA** | January – February 2012    UK | 7 | 26.3  (6.3) | Mean of empirical serial intervals from study cases with reported dates of clinical presentation | Hospital outbreak in paediatric burns centre, 2010    Index case and other cases laboratory confirmed after date of presentation | Patel, 2013 [[57]](https://paperpile.com/c/YpND2q/hK8FK) |
|  | 2009  UK | 12 | 8.2 (5.7) | Mean of empirical serial intervals from study cases with reported dates of isolation. | NICU outbreak, UK, 2009    Index case bacteraemia and other cases laboratory confirmed | Koser, 2012 [[58]](https://paperpile.com/c/YpND2q/eY1D4) |
|  | July 2012    Korea | 7 | 17.5 (2.1) | Mean of empirical serial intervals from study cases with reported dates of isolation. | NICU outbreak, South Korea, 2012    Index case and other cases laboratory confirmed | Lee, 2014 [[59]](https://paperpile.com/c/YpND2q/hP0fs) |
| ***K. pneumoniae*** | November – December 2000    Netherlands | 8 | 60.1 (15.5) | Mean of empirical serial intervals derived from first MRKP (multi resistant *K.pneumoniae*) isolation date in index case and secondary cases. | Hospital outbreak    Index case and other cases laboratory confirmed | Van’t Veen, 2005 [[69]](https://paperpile.com/c/YpND2q/MrMLa) |
|  | December 2003 – August 2004    France | 8 | 64.7 (49.5) | Mean of empirical serial intervals derived from first MRKP isolation date in index case, secondary cases and tertiary cases. | Hospital outbreak    Index case and other cases laboratory confirmed | Kassis-Chikhani, 2006 [[70]](https://paperpile.com/c/YpND2q/4XkrH) |
|  | September – November 1997    UK | 11 | 63.2 (12.9) | Mean of empirical serial intervals derived from first MRKP isolation date in index case and secondary cases. No tertiary transmission assumed. | NICU outbreak    Index case and other cases laboratory confirmed | Macrae, 2001 [[71]](https://paperpile.com/c/YpND2q/wxQ5l) |
| ***S. pneumoniae*** | November 2000    USA | 12 | 5.7 (1.1) | Mean of empirical serial intervals derived from first isolation date in index case and secondary cases. No tertiary transmission assumed. | Outbreak in Marine training base    Index case and other cases laboratory confirmed | Crum, 2003 [[79]](https://paperpile.com/c/YpND2q/PIq9c) |
|  | August 2012    UK | 15 | 1.9 (0.8) | Mean of empirical serial intervals from symptom onset of reported study cases. No tertiary transmission assumed. | Outbreak in nursing home    Index case and other cases probable or confirmed | Thomas, 2015 [[80]](https://paperpile.com/c/YpND2q/AKjPk) |
|  | April 2013    Japan | 7 | 17.5 (4.1) | Mean of empirical serial intervals from reported study cases. No tertiary transmission | Outbreak in nursing home, 2013, Japan    Index case and other cases, symptom onset in laboratory confirmed cases | Kuroki, 2014 [[81]](https://paperpile.com/c/YpND2q/9tjAN) |
| ***M. tuberculosis*** | 1993 – 1996    Netherlands | 69 | 273.7  (245.1) | Mean of empirical serial intervals from reported clinical cases | Reported cases in The Netherlands from 1993 to 1996    Transmission identified by identical DNA footprints and epidemiological confirmation of contact | Ten Asbroek, 1999 [[89]](https://paperpile.com/c/YpND2q/XAyPK) |
|  | 1997 – 2010    Germany | 86 | 365  (496.4) | Bayesian inference of generation time distribution from transmission tree reconstruction | Longitudinal study in Hamburg, Germany from 1997 to 2010    WGS and surveillance data used to infer likely transmission pairs | Didelot, 2017 [[12]](https://paperpile.com/c/YpND2q/rg2QE)  Roetzer, 2013 [[90]](https://paperpile.com/c/YpND2q/0hBDG) |
| ***S. sonnei*** | June 1982    USA | 17 | 8.5  (3) | Time from exposure to symptoms. Mean incubation period of 2.4 days (Makintubee, 1987)    ‘Infection usually lasts for 4–7 days and is self limiting’ (ECDC, 2016)    ‘Most patients recover uneventfully within seven to ten days’ (WHO, 2005) | Outbreak associated with contaminated lake    Given a mean incubation period of 2.4 days, a reported infectious period of 4-7 days and a reported time from infection to recovery of 7-10 days, a mean generation time of 8.5 days was chosen. No data on the standard deviation was available, however given the narrow ranges provided on infectious periods and times to recovery, a standard deviation of 3 days was chosen | Makintubee, 1987 [[95]](https://paperpile.com/c/YpND2q/QGIJD)  WHO, 2005  [[96]](https://paperpile.com/c/YpND2q/VUjRO)  ECDC, 2016 [[97]](https://paperpile.com/c/YpND2q/dpzot) |
| ***C. difficile*** | January 1998 - December 2009    Canada | 8 | 20.1  (14.9) | Mean of empirical interval between symptom onset of household transmission pairs | Community outbreak. 2222 patients diagnosed with *C. dificile* infection (CDI) identified from a hospital database. Pairs in the same household identified and verified by telephone | Pepin, 2012 [[101]](https://paperpile.com/c/YpND2q/xlGkX) |
|  | September 2007 - March 2010    UK | 218 | 28 | Sum of median incubation period and median time from symptom onset to onward infection for most plausible transmission links | Outbreak in hospital. 1276 patients diagnosed with CDI and genotyped by multilocus sequence typing (MLST). 218 putative transmission pairs identified by sequence type homology and ward-based contact | Walker, 2012 [[102]](https://paperpile.com/c/YpND2q/uR3U7) |
