# Supplementary material for: When are pathogen genome sequences informative of transmission events?
Source: PLoS Pathog. 2018 Feb 8;14(2):e1006885. doi: 10.1371/journal.ppat.1006885 (PMC5821398; doi:10.1371/journal.ppat.1006885)
Supplement: S2 Table — (DOCX) [file ppat.1006885.s005.docx]

### **S2 Table. Mutation rates**

| **Pathogen** | **Mean mutation rate of nucleotides per site per year (credible intervals)** | **First author, year [reference]** |
| --- | --- | --- |
| **EBOV** | 1.30 x 10^-3^ | Hoenen, 2014 [[31]](https://paperpile.com/c/YpND2q/2kcO) |
|  | 0.90 x 10^-3^ | Gire, 2014 [[32]](https://paperpile.com/c/YpND2q/XZYa) |
|  | 1.23 x 10^-3^ (1.04, 1.41) | Tong, 2015 [[33]](https://paperpile.com/c/YpND2q/I2TQ) |
| **MERS-CoV** | 1.00 x 10^-3^ (0.68, 1.30) | Cauchemez, 2014 [[35]](https://paperpile.com/c/YpND2q/DbA3R) |
|  | 0.63 x 10^-3^ (0.11, 0.14) | Cotten, 2013 [[38]](https://paperpile.com/c/YpND2q/IgJqb) |
|  | 1.12 x 10^-3^ (0.88, 1.37) | Cotten, 2014 [[39]](https://paperpile.com/c/YpND2q/HnANf) |
| **SARS-CoV** | 2.08 x 10^-3^ | Vega, 2004 [[44]](https://paperpile.com/c/YpND2q/mr36J) |
|  | 2.38 x 10^-3^ | Zhao, 2004 [[45]](https://paperpile.com/c/YpND2q/uKFpl) |
|  | 8.04 x 10^-3^ | Wu, 2003 [[46]](https://paperpile.com/c/YpND2q/WTJV0)) |
| **Influenza A (H1N1)** | 3.66 x 10^-3^ (0.61, 6.58) | Smith, 2009 [[53]](https://paperpile.com/c/YpND2q/Jjkd3) |
|  | 5.02 x 10^-3^ (4.17, 5.95) | Rambaut and Holmes, 2009 [[54]](https://paperpile.com/c/YpND2q/qWXyN) |
| **MRSA** | 1.22 x 10^-6^ (0.60, 1.86) | Uhlemann, 2014 [[60]](https://paperpile.com/c/YpND2q/6KHOX) |
|  | 1.30 x 10^-6^ (1.20, 1.40) | Holden, 2013 [[61]](https://paperpile.com/c/YpND2q/Bbt03) |
|  | 1.40 x 10^-6^ (1.04, 1.80) | McAdam, 2012 [[62]](https://paperpile.com/c/YpND2q/E2Jl6) |
|  | 3.30 x 10^-6^ (2.50, 4.00) | Harris, 2010 [[63]](https://paperpile.com/c/YpND2q/j6H61) |
|  | 1.21 x 10^-6^ | Harris, 2013 [[64]](https://paperpile.com/c/YpND2q/kL7VG) |
|  | 2.99 x 10^-6^ | Nubel, 2013 [[61]](https://paperpile.com/c/YpND2q/Bbt03) |
| ***K. pneumoniae*** | 1.90 x 10^-6^ (1.10, 2.90) | Mather, 2015 [[72]](https://paperpile.com/c/YpND2q/XnzEH) |
|  | 2.70 x 10^-6^ (0.68, 5.00) | Chung The 2015, [[22]](https://paperpile.com/c/YpND2q/4ywTh) |
|  | 3.65 x 10^-6^ | De Champs, 2004 [[74]](https://paperpile.com/c/YpND2q/KqPjF) |
| ***S. pneumoniae*** | 1.27 x 10^-6^ | Stevens and Sebert, 2011 [[82]](https://paperpile.com/c/YpND2q/lJ9d4) |
|  | 3.12 x 10^-6^ | Henderson-Begg, 2006 [[83]](https://paperpile.com/c/YpND2q/CaIZ0) |
|  | 1.57 x 10^-6^ | Croucher, 2011 [[84]](https://paperpile.com/c/YpND2q/nEom7) |
| ***M. tuberculosis*** | 0.50 (SNPs/genome/year) | Walker, 2013 [[18]](https://paperpile.com/c/YpND2q/rDe5s) |
|  | 0.26 (SNPs/genome/year) | Guerra-Assuncao, 2015 [[91]](https://paperpile.com/c/YpND2q/npKjK) |
| ***S. sonnei*** | 6.0 x 10^-7^ (5.2, 6.7) | Holt, 2012 [[98]](https://paperpile.com/c/YpND2q/JYwM) |
| ***C. difficile*** | 3.2 x 10^-7^ (1.3, 5.3) | Didelot, 2012 [[23]](https://paperpile.com/c/YpND2q/hH5p5) |
