# Supplementary material for: When are pathogen genome sequences informative of transmission events?
Source: PLoS Pathog. 2018 Feb 8;14(2):e1006885. doi: 10.1371/journal.ppat.1006885 (PMC5821398; doi:10.1371/journal.ppat.1006885)
Supplement: S3 Table — (DOCX) [file ppat.1006885.s006.docx]

### **S3 Table. Genome lengths**

| **Pathogen** | **Accession number** | **Core genome length (base pairs)** | **First author, year [reference]** |
| --- | --- | --- | --- |
| **EBOV** | KJ660346 | 18959 | Baize, 2014 [[34]](https://paperpile.com/c/YpND2q/9qEC) |
|  | KP096420 | 18958 | Hoenen, 2014 [[31]](https://paperpile.com/c/YpND2q/2kcO) |
|  | KP096421 | 18958 | Hoenen, 2014 [[31]](https://paperpile.com/c/YpND2q/2kcO) |
|  | KP096422 | 18958 | Hoenen, 2014 [[31]](https://paperpile.com/c/YpND2q/2kcO) |
| **MERS-CoV** | KF186567 | 30117 | Cotten, 2013 [[38]](https://paperpile.com/c/YpND2q/IgJqb) |
|  | KF186566 | 30113 | Cotten, 2013 [[38]](https://paperpile.com/c/YpND2q/IgJqb) |
|  | KF600547 | 30115 | Cotten, 2013 [[38]](https://paperpile.com/c/YpND2q/IgJqb) |
|  | JX869059.2 | 30119 | De Groot, 2013 [[40]](https://paperpile.com/c/YpND2q/7WQl9) |
|  | KC164505.2 | 30111 | De Groot, 2013 [[40]](https://paperpile.com/c/YpND2q/7WQl9) |
| **SARS-CoV** | AY278741 | 29727 | Rota, 2003 [[47]](https://paperpile.com/c/YpND2q/cMEYD) |
|  | AY283794 | 29711 | Ruan, 2003 [[48]](https://paperpile.com/c/YpND2q/P9x5T) |
|  | AY283792 | 29705 | Ruan, 2003 [[48]](https://paperpile.com/c/YpND2q/P9x5T) |
|  | AY283796 | 29711 | Ruan, 2003 [[48]](https://paperpile.com/c/YpND2q/P9x5T) |
| **Influenza A (H1N1)** | KC781896-03 | 13158 | Influenza Virus Resource [[55]](https://paperpile.com/c/YpND2q/YQenT) |
|  | HM569667-74 | 13158 | Influenza Virus Resource [[55]](https://paperpile.com/c/YpND2q/YQenT) |
|  | JX625635-42 | 13158 | Influenza Virus Resource [[55]](https://paperpile.com/c/YpND2q/YQenT) |
| **MRSA** | BX571856 | 2902619 | Holden, 2004 [[65]](https://paperpile.com/c/YpND2q/XQOTo) |
|  | HE681097 | 2832299 | Holden, 2013 [[61]](https://paperpile.com/c/YpND2q/Bbt03) |
|  | FR821777 | 2762785 | Holt, 2011 [[66]](https://paperpile.com/c/YpND2q/d8f00) |
|  | NC_007793 | 2872769 | Diep, 2006 [[67]](https://paperpile.com/c/YpND2q/r0HFg) |
| ***K. pneumoniae*** | CP003200 | 5332752 | Liu, 2012 [[75]](https://paperpile.com/c/YpND2q/GqsBQ) |
|  | HF536482 | 5324709 | Fookes, 2013 [[76]](https://paperpile.com/c/YpND2q/vrJ5T) |
|  | CP002910 | 5259571 | Shin, 2012 [[77]](https://paperpile.com/c/YpND2q/0QrlE) |
| ***S. pneumoniae*** | AE005672 | 2160842 | Tettelin, 2001 [[85]](https://paperpile.com/c/YpND2q/4GSHs) |
|  | NC_014491 | 2130580 | Camilli, 2011 [[86]](https://paperpile.com/c/YpND2q/wlTak) |
|  | *Published estimate* | 2088534 | Donati, 2010 [[87]](https://paperpile.com/c/YpND2q/nBrSz) |
| ***M. tuberculosis*** | AL123456.2 | 4411532 | Cole, 1998 [[92]](https://paperpile.com/c/YpND2q/QwSab) |
|  | NC_018143.2 | 4411709 | Camus, 2002 [[93]](https://paperpile.com/c/YpND2q/u57qn) |
| ***S. sonnei*** | NC_007384 | 4825265 | Yang, 2005 [[99]](https://paperpile.com/c/YpND2q/yufs) |
| ***C. difficile*** | AM180355.1 | 4290252 | Sebaihia, 2006 [[103]](https://paperpile.com/c/YpND2q/OhWEa) |
