# Supplementary material for: When are pathogen genome sequences informative of transmission events?
Source: PLoS Pathog. 2018 Feb 8;14(2):e1006885. doi: 10.1371/journal.ppat.1006885 (PMC5821398; doi:10.1371/journal.ppat.1006885)
Supplement: S4 Table — (DOCX) [file ppat.1006885.s007.docx]

### **S4 Table. Basic reproduction number R_0_**

| **Pathogen** | **Study period and location** | **Basic reproduction**  **number R_0_** | **Study method** | **First author, year [reference]** |
| --- | --- | --- | --- | --- |
| **EBOV** | November 2014    Guinea | 1.7 | Inference from time series of incidence of cases and from estimates of the serial interval | WHO ERT, 2014 [[28]](https://paperpile.com/c/YpND2q/ilQz) |
|  | Liberia | 1.8 | “” | “” |
|  | Sierra Leone | 2.0 | “” | “” |
| **MERS-CoV** | 2012 – 2013    France, Jordan, Saudi Arabia, UK | 1.2 | Identified clusters of infection, and determined probabilistically the number of secondary infections caused by the index case of each cluster using the generation time distribution and timing of cases | Cauchemez, 2014 [[35]](https://paperpile.com/c/YpND2q/DbA3R) |
| **SARS-CoV** | 2003    Hong Kong | 2.7 | Fitting a stochastic metapopulation compartmental model to incidence data on 1512 cases | Riley, 2003 [[49]](https://paperpile.com/c/YpND2q/8nfOx) |
| **Influenza A (H1N1)** | N/A | 1.5 | A systematic review of estimates of R_0_. 24 studies were selected, with a range of 1.2 – 2.3, with a median of 1.5 | Boelle, 2011 [[56]](https://paperpile.com/c/YpND2q/N0Gy6) |
| **MRSA** | January 2004 – December 2006    USA | 1.3 | Fitting an age-structured compartmental model to time-series skin and soft tissue infections in children, in the presence of infection control measures | Wang, 2013 [[68]](https://paperpile.com/c/YpND2q/pyEkc) |
| ***K. pneumoniae*** | May 2009 – June 2010    Greece | 2.0 | Fitting the Ross-Macdonald model for vector-borne diseases to epidemiological and infection control data collected in a surgical unit of a tertiary-care hospital | Sypsa, 2012 [[78]](https://paperpile.com/c/YpND2q/IHu14) |
| ***S. pneumoniae*** | September 2001 – May 2002    Finland | 1.4 | Fitting a time event history model to longitudinal data on children and family members in day care cohorts | Hoti, 2009 [[88]](https://paperpile.com/c/YpND2q/c9bLD) |
| ***M. tuberculosis*** | Unknown | 1.15 | Bayesian inference of transmission network from sampling times and whole genome sequences. | Didelot, 2014 [[11]](https://paperpile.com/c/YpND2q/TU6Ss) |
|  | Unknown | 1.60 | Fitting a compartmental model of pulmonary TB to treatment results of six countries | Dye, 2000 [[94]](https://paperpile.com/c/YpND2q/TehYE) |
| ***S. sonnei*** | 1967 – 2007    USA | 1.1 | Fitting a time-series SIR model to county level shigellosis data. Mean of the R_0_ estimates with the highest log-likelihood for each county evaluated | Joh, 2013 [[100]](https://paperpile.com/c/YpND2q/Om4R) |
| ***C. difficile*** | January – December 2008    USA | 1.07 | Fitting a compartmental model of *C. difficile* transmission to a hospital data set of 11046 patients diagnosed with CDI by stool toxin analysis | Lanzas, 2011 [[104]](https://paperpile.com/c/YpND2q/DXD4d) |
|  | February 1999 – January 2000    Sweden | 1.9 | Analysis of 330 isolates diagnosed by stool toxin analysis; secondary cases identified by PCR ribotyping | Noren, 2004 [[105]](https://paperpile.com/c/YpND2q/6a38I) |
