# Supplementary material for: When are pathogen genome sequences informative of transmission events?
Source: PLoS Pathog. 2018 Feb 8;14(2):e1006885. doi: 10.1371/journal.ppat.1006885 (PMC5821398; doi:10.1371/journal.ppat.1006885)
Supplement: S5 Table — (DOCX) [file ppat.1006885.s008.docx]

### **S5 Table. Transmission divergence and its effect on outbreak reconstruction for different pathogens.**

| **Pathogen** | **Transmission divergence:**  **mean (IQR)** | | **Number unique sequences / outbreak size:**  **mean (IQR)** | | **Change in accuracy of outbreak reconstruction: mean (IQR)** | |
| --- | --- | --- | --- | --- | --- | --- |
|  | ***outbreaker*** | ***phybreak*** | ***outbreaker*** | ***phybreak*** | ***outbreaker*** | ***phybreak*** |
| **EBOV** | 0.79 (0.71, 0.86) | 1.46 (1.28, 1.61) | 0.51 (0.47, 0.55) | 0.64 (0.59, 0.69) | 0.36 (0.31, 0.40) | 0.32 (0.27, 0.38) |
| **MERS-CoV** | 0.82 (0.70, 0.94) | 1.40 (1.23, 1.56) | 0.54 (0.49, 0.57) | 0.64 (0.59, 0.70) | 0.38 (0.30, 0.45) | 0.29 (0.23, 0.35) |
| **SARS-CoV** | 2.72 (2.52, 2.91) | 4.83 (4.63, 5.01) | 0.89 (0.87, 0.91) | 0.98 (0.97, 0.99) | 0.78 (0.75, 0.82) | 0.60 (0.56, 0.65) |
| ***Influenza* A (H1N1)** | 0.45 (0.39, 0.50) | 0.81 (0.71, 0.92) | 0.36 (0.32, 0.41) | 0.45 (0.41, 0.48) | 0.29 (0.23, 0.36) | 0.28 (0.22, 0.34) |
| **MRSA** | 0.23 (0.18, 0.29) | 0.41 (0.31, 0.50) | 0.21 (0.18, 0.25) | 0.26 (0.23, 0.30) | 0.14 (0.08, 0.19) | 0.14 (0.09, 0.19) |
| ***K. pneumoniae*** | 2.02 (1.90, 2.13) | 3.40 (3.18, 3.63) | 0.83 (0.81, 0.86) | 0.93 (0.92, 0.95) | 0.74 (0.71, 0.78) | 0.60 (0.55, 0.64) |
| ***S. pneumoniae*** | 0.08 (0.06, 0.10) | 0.13 (0.07, 0.17) | 0.10 (0.07, 0.12) | 0.10 (0.07, 0.13) | 0.12 (0.05, 0.19) | 0.09 (0.02, 0.14) |
| ***M. tuberculosis*** | 0.31 (0.27, 0.37) | 0.58 (0.46, 0.67) | 0.27 (0.23, 0.30) | 0.35 (0.30, 0.39) | 0.19 (0.14, 0.24) | 0.21 (0.16, 0.26) |
| ***S. sonnei*** | 0.06 (0.03, 0.08) | 0.09 (0.04, 0.14) | 0.08 (0.06, 0.10) | 0.09 (0.06, 0.10) | 0.09 (0.02, 0.14) | 0.06 (0.00, 0.12) |
| ***C. difficile*** | 0.10 (0.07, 0.12) | 0.18 (0.12, 0.23) | 0.11 (0.08, 0.13) | 0.13 (0.10, 0.17) | 0.08 (0.04, 0.13) | 0.09 (0.05, 0.12) |

Transmission divergence is defined as the number of mutations separating pathogen genomes sampled from transmission pairs. For each pathogen, 100 outbreaks were simulated using both *outbreaker* and *phybreak* and the transmission divergence calculated as the mean number of mutations between transmission pairs. The proportion of unique WGS was also determined for each outbreak. Each outbreak was then reconstructed using *outbreaker* or *phybreak*, and the accuracy of outbreak reconstruction defined as the proportion of correctly assigned ancestries in the consensus transmission tree. The change in accuracy represents the difference in accuracy of outbreak reconstruction upon including WGS, relative to using only sampling times.
