# Supplementary material for: When are pathogen genome sequences informative of transmission events?
Source: PLoS Pathog. 2018 Feb 8;14(2):e1006885. doi: 10.1371/journal.ppat.1006885 (PMC5821398; doi:10.1371/journal.ppat.1006885)
Supplement: S6 Table — (DOCX) [file ppat.1006885.s009.docx]

### **S6 Table. Proportion of unique WGS collected in an outbreak setting.**

| **Pathogen** | **Study period and location** | **Unique sequences / number of sequences** | **Proportion of unique sequences** | **Data description** | **First author, year [reference]** |
| --- | --- | --- | --- | --- | --- |
| **EBOV** | 2014  Sierra Leone | 31/77 | 0.40 | Gire *et al.* collected WGS from 78 cases in the early stages of the outbreak in Sierra Leone, representing over 70% of known cases at the time. The aligned sequences were downloaded from the study by Famulare *et al.*, and the number of unique sequences determined. | Gire, 2014 [[32]](https://paperpile.com/c/YpND2q/XZYa)  Famulare and Hu, 2015 [[116]](https://paperpile.com/c/YpND2q/4DFA) |
|  | December 2014 - September 2015  Mamusa, Sierra Leone | 7/9 | 0.78 | 554 WGS were collected in from Ebola Virus Disease cases across Sierra Leone. Three transmission clusters of highly related viral sequences were described by Arias *et al.*, and the number of unique sequences determined by visual inspection of the phylogenies.  Mamusa cluster: see Arias *et al.*, Figure 3 | Arias, 2016 [[117]](https://paperpile.com/c/YpND2q/nyIR) |
|  | Tonkolilli and Magazie Wharf , Sierra Leone | 22/24 | 0.92 | Tonkolilli and Magazine Wharf cluster: see Arias *et al.*, Figure 4 | “” |
|  | Kambia, Sierra Leone | 10/17 | 0.59 | Kambia cluster: see Arias *et al.*, Figure 5 | “” |
| **MERS-CoV** | 2013 - 2014  United Arab Emirates | 9/15 | 0.60 | Full MERS-CoV genome sequences were collected from 19 cases, alongside extensive epidemiological data. Six transmission clusters involving 15 transmission events were identified, and the number of nucleotide differences between these pairs described (Paden *et al.*, Table 1) | Paden, 2017 [[118]](https://paperpile.com/c/YpND2q/sck7) |
| **SARS-CoV** | 2003  Singapore | 12/12 | 1.00 | Ruan *et al.* collected full SARS-CoV genomes from 13 cases in the early stage of the outbreak. The pairwise number of mutations separating these cases were described by *Jombart* et al. (Figure S13), with no identical sequences observed. | Ruan, 2003 [[48]](https://paperpile.com/c/YpND2q/P9x5T)  Jombart, 2014 [[5]](https://paperpile.com/c/YpND2q/Giqib) |
| **Influenza A** | 2014 - 2015  Bavaria, Germany | 2/6 | 0.33 | 50 Influenza A WGS were produced, and 6 direct transmission events supported by both epidemiological and genetic data identified. The numbers of mutations separating these pairs are described in the body of the text. | Meinel, 2017 [[119]](https://paperpile.com/c/YpND2q/0i6X) |
| ***K. pneumoniae*** | 2011  USA | 11/18 | 0.61 | WGS were collected from 18 patients in an outbreak of carbapenem-resistant *K. pneumoniae*. The genetic diversity among these sequences is described in Figure 2B, from which the number of unique sequences were identified.  The average generation time in the outbreak was determined by calculating the time between positive culture dates (Snitkin *et al.*, Table 1) of putative transmission pairs (Snitkin *et al.*, Figure 3). | Snitkin, 2012 [[106]](https://paperpile.com/c/YpND2q/tD3c) |
| ***M. tuberculosis*** | 1994 - 2011  UK | 15/38 | 0.40 | *M. tuberculosis* WGS were collected from 254 patients in a retrospective sequencing study. The genetic distance between individuals in 38 known, recent household transmission events was determined (Walker *et al.*, Figure 2A) and the number of unique sequences counted. | Walker, 2013 [[18]](https://paperpile.com/c/YpND2q/rDe5s) |
|  | 1997 - 2010  Germany | 12/31 | 0.39 | 86 WGS were collected during a large *M. tuberculosis* outbreak of the Haarlem strain, and definite transmission between 31 cases revealed by contact tracing. 19 of these sequences were genetically identical. | Roetzer, 2013 [[90]](https://paperpile.com/c/YpND2q/0hBDG) |
| ***C. difficile*** | September 2006 - June 2010  Oxfordshire, UK | 93/333 | 0.28 | WGS were collected from 1223 cases of *C. difficile* infection arising over four years. 333 cases had a plausible infector in this dataset, defined as a case with a genetic distance of 3 or fewer mutations to the putative infectee. Among these, there existed 93 unique sequences. | Eyre, 2013 [[17]](https://paperpile.com/c/YpND2q/uTCZj) |
